# DEMIGOD: A Low-Cost Microcontroller-Based Closed-Loop System Integrating Nanoengineered Sweat-Based Glucose Monitoring and Controlled Transdermal Nanoemulsion Release of Hypoglycemic Treatment with a Software Application for Noninvasive Personalized Diabetes Care

**DOI:** 10.3390/mi15070887

**Published:** 2024-07-06

**Authors:** Vasiliki Fiska, Eirini Papanikolaou, Michaela Patila, Mamas I. Prodromidis, Maria G. Trachioti, Eleni I. Tzianni, Konstantinos Spyrou, Pantelis Angelidis, Markos G. Tsipouras

**Affiliations:** 1 Department of Electrical and Computer Engineering, University of Western Macedonia, 50100 Kozani, Greece; dece00073@uowm.gr (V.F.); paggelidis@uowm.gr (P.A.); 2 Laboratory of Physiology, Faculty of Medicine, University of Ioannina, 45110 Ioannina, Greece; eirini.papanikolaou0@gmail.com; 3 Biotechnology Laboratory, Department of Biological Applications and Technologies, University of Ioannina, 45110 Ioannina, Greece; michaelapatila@gmail.com; 4 Laboratory of Analytical Chemistry, University of Ioannina, 45110 Ioannina, Greece; mprodrom@uoi.gr (M.I.P.); trachioti93@gmail.com (M.G.T.); el.tzianni@gmail.com (E.I.T.); 5 Department of Materials Science and Engineering, University of Ioannina, 45110 Ioannina, Greece; konstantinos.spyrou1@gmail.com

**Keywords:** diabetes mellitus, glucose monitoring, noninvasive, closed loop, software application, hypoglycemic treatment

## Abstract

This study endeavored to design and develop an innovative closed-loop diagnostic and therapeutic system with the following objectives: (a) the noninvasive detection of glucose concentration in sweat utilizing nanonengineered screen-printed biosensors; (b) the management of measured data through a specialized computer system comprising both hardware and software components, thereby enabling the precise control of therapeutic responses via a patch-based nanomedicine delivery system. This initiative addresses the significant challenges inherent in the management of diabetes mellitus, including the imperative need for glucose-level monitoring to optimize glycemic control. Leveraging chronoamperometric results as a foundational dataset and the in vivo hypoglycemic activity of nanoemulsion formulations, this research underscores the efficacy and accuracy of glucose concentration estimation, decision-making mechanism responses, and transdermal hypoglycemic treatment effects, within the proposed system.

## 1. Introduction

Diabetes mellitus (DM), a chronic and multifaceted metabolic disorder, is represented by sustained hyperglycemia arising from factors such as insulin resistance, inadequate insulin secretion, or a combination thereof [1]. Insulin, a pivotal hormone synthesized by the pancreas, regulates carbohydrate and fat metabolism, facilitating glucose uptake into cells for energy production [2]. Disruption of this physiological mechanism results in hyperglycemia, a major clinical manifestation of the diabetic state, leading to diverse complications in the absence of adequate management [3].

Moreover, deficiencies in insulin or resistances to its effects are linked with irregularities in lipid and protein metabolism, as well as disruptions in mineral and electrolyte balance [4]. The majority of DM patients are classified into two primary categories: Type 1 DM (T1DM), caused by an absolute or a near absolute deficiency of insulin, or Type 2 DM (T2DM), characterized by insulin resistance with an inadequate compensatory increase in insulin secretion [5]. In addition, women who develop DM during their pregnancy are classified as having gestational diabetes; the various uncommon and diverse types of diabetes arise from infections, drugs, endocrinopathies, pancreatic destruction, and genetic defects are classified separately as ”Other Specific Types” [6]. DM imposes a significant global health burden [7], featuring among the top 10 leading causes of death globally according to the World Health Organization (WHO) (source: https://www.who.int/news-room/fact-sheets/detail/the-top-10-causes-of-death (accessed on 24 April 2024), WHO—The top 10 causes of death), with its increasing prevalence and associated complications.

Despite advances in DM management, achieving optimal glycemic control remains a challenge for many individuals, necessitating continuous monitoring and personalized treatment approaches [8]. Conventional glucose monitoring (GM) methods, such as fingerstick blood glucose testing, while effective, are often intrusive and inconvenient, leading to suboptimal adherence and outcomes [9]. In recent years, there has been growing interest in the development of noninvasive or minimally invasive monitoring technologies to enhance the management of DM [10]. Among these, sweat-based glucose monitoring (SGM) [11] has emerged as a promising approach due to its noninvasive nature and potential for continuous glucose monitoring (CGM) without the need for frequent blood sampling [12]. Sweat, a biofluid rich in biomarkers, offers valuable insights into an individual’s physiological state, including glucose levels closely mirroring those found in the bloodstream, making it an attractive target for monitoring applications [13]. The correlation between blood glucose levels and sweat glucose levels has been a focus of research for noninvasive glucose monitoring in DM patients. According to Sankhala D. et al. [11], measuring blood glucose accurately is challenging due to the low mean glucose level in the human body (4 g) and the risks associated with hypoglycemia (below 70 mg/dL) and hyperglycemia (above 130 mg/dL). GM devices must also avoid interference from glucose oxidase and other enzymes, which can cause secondary reactions. Studies, including those by Moyer et al. [14], have demonstrated a strong correlation between blood and sweat glucose levels, establishing the potential of sweat glucose as a noninvasive monitoring marker.

Evolutionx in nanoengineering have further propelled the development of sweat-based sensors with enhanced sensitivity, selectivity, and wearability [15]. Nanostructured materials and fabrication techniques have enabled the design of highly sensitive and minimally invasive sensors capable of detecting glucose concentrations in sweat with high accuracy and precision [16]. These nanoengineered sensors hold great promise for revolutionizing DM management by providing real-time CGM levels in a convenient and unobtrusive manner.

The integration of screen-printed electrode (SPE) technology has significantly contributed to the advancement of sweat-based sensors [17]. SPE sensors offer several advantages, including low-cost production, flexibility, and ease of customization [18]. By leveraging the unique properties of SPEs, such as their high surface-area-to-volume ratio and excellent electrochemical performance, many innovative sensor designs have been developed that enhance the sensitivity and reliability of glucose detection in sweat [19]. Additionally, SPEs can be tailored to detect multiple analytes simultaneously, expanding the capabilities of sweat-based sensor platforms for comprehensive health monitoring [20]. This integration of SPE technology further enhances the potential of sweat-based sensors to revolutionize DM management, offering continuous, noninvasive monitoring solutions that empower individuals to proactively manage their health.

In parallel, the field of transdermal drug delivery has witnessed significant advancements, offering new opportunities for the controlled and targeted delivery of hypoglycemic agents [21]. Nanoemulsions, colloidal dispersions of nanoscale droplets, have emerged as promising vehicles for transdermal drug delivery due to their ability to encapsulate hydrophobic and hydrophilic drugs, enhance drug solubility, and improve skin permeation [22]. By incorporating hypoglycemic agents into nanoemulsion formulations, controlled transdermal delivery systems can be developed to enable the on-demand administration of insulin or other antidiabetic medications based on real-time GM data [23].

DM management has transitioned into a pivotal phase characterized by the proliferation of increasingly sophisticated digital interventions [24]. In recent years, the healthcare domain has experienced a notable transformation through the integration of web-based mobile technology, particularly in the realm of chronic disease management [25]. In addressing the exigencies of DM, a condition necessitating continual surveillance and prompt interventions, web applications have assumed a pivotal role, evolving into indispensable tools [26]. The adoption of these applications has revolutionized the conventional framework of DM management, enabling patients and healthcare providers alike to monitor glucose levels, dietary patterns, and physical activity, while receiving tailored feedback to augment the self-management of the condition [27].

Closed-loop systems, often described as “artificial pancreas” systems, represent a cutting-edge approach in DM management, particularly in the context of wearable or portable devices [28]. These systems utilize a combination of GM technology and automated insulin delivery (AID) systems to regulate blood glucose levels [29,30]. The GM component monitors the glucose levels in interstitial fluid, providing a stream of data to the control algorithm [31]. Simultaneously, the AID system administers insulin or other glucose-lowering medications based on the GM readings, effectively mimicking the function of the pancreas in response to fluctuating glucose levels [32]. By integrating these components into a wearable or portable device, individuals with DM can benefit from seamless and personalized glucose control, minimizing the risk of hypoglycemia and enhancing their quality of life.

## 2. Materials and Methods

**Figure 1:**
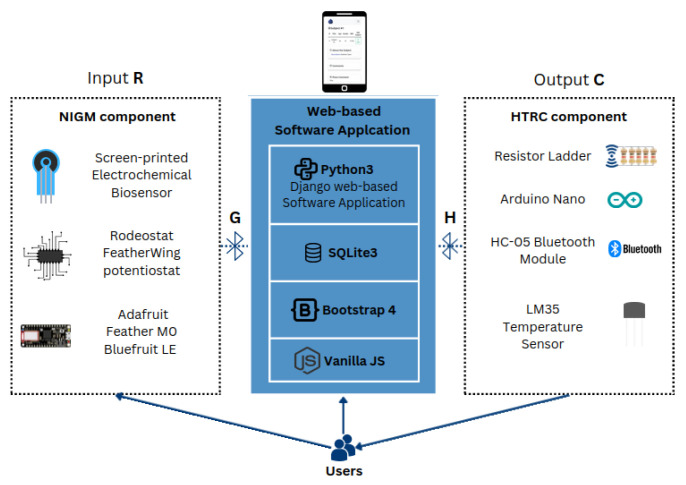
Diagram of the DEMIGOD nanoengineered noninvasive closed-loop system for GM and treatment.

A novel nanoengineered, noninvasive, closed-loop system that integrates noninvasive SGM with the controlled transdermal nanoemulsion release of hypoglycemic treatment connected with a web-based software application for personalized DM care [33] is presented in this manuscript. Figure 1 presents an illustrative depiction of the proposed closed-loop system alongside its associated elements. The input variable ”R” is acquired through the noninvasive glucose monitoring (NIGM) component, utilizing a sweat-based solution for GM. The component denoted by ”G” embodies the process dynamics of the system’s web-based software application, encapsulating its operational intricacies and functional behaviors. Conversely, the entity represented by ”H” delineates the feedback and control mechanisms subsequent to the output process denoted by ”C” within the hypoglycemic treatment release circuit (HTRC) component. Therefore, individuals can easily access their SGM records in real time, facilitating timely interventions and adjustments in hyperglycemic treatment or lifestyle choices. The presented integration enables DM monitoring and personalized feedback, empowering individuals to make informed decisions and take proactive steps toward better health outcomes, enhance patient adherence, and ultimately reduce the risk of DM-related complications.

### 2.1. Noninvasive Sweat-Based Glucose Monitoring

SGM is a no-invasive approach to GM that offers several advantages over traditional blood-based methods. The principle behind SGM lies in the correlation between the glucose levels in sweat and blood. Glucose diffuses passively from the blood into the interstitial fluid, where it can subsequently be detected in sweat [34]. This process is influenced by factors such as hydration status, skin temperature, and sweat rate.

Various techniques have been developed to measure the glucose levels in sweat, including electrochemical sensors, colorimetric assays, and spectroscopic methods [35]. These sensors typically consist of a sensing element that selectively interacts with glucose molecules, generating a measurable signal proportional to the glucose concentration [36]. Advances in sensor technology have led to the development of wearable devices capable of real-time monitoring, offering individuals with DM a convenient and continuous means of GM without the need for frequent fingerstick testing [37].

Nanoengineering has played a crucial role in the development of sensitive and selective sensors for SGM [38]. Nanomaterials such as carbon nanotubes [39], graphene [40], and metal [41] offer unique properties that enhance sensor performance, including high surface-to-volume ratio, excellent electrical conductivity, and biocompatibility. Therefore, by incorporating nanomaterials into sensor designs, sensitivity, selectivity, and response time can be improved, enabling the detection of glucose at clinically relevant concentrations.

#### 2.1.1. Screen-Printed Electrochemical Biosensor

The electrochemical determination of glucose has been studied almost exclusively with amperometric enzyme biosensors, since their analytical behavior is superior compared to that of other instrumental techniques [42]. The enzyme of choice in the majority of these biosensors is glucose oxidase (GOx), and the reactions in the presence of glucose are described by the following chemical equation [43]:

$$\left[GOx-FADH_{2}\right]+O_{2}\to \left[GOx-FAD\right]+H_{2}O_{2}$$

The DEMIGOD system incorporates a nanoengineered screen-printed electrochemical biosensor, as designed and manifactured by Alatzoglou C. et al. [44]. This biosensor integrates the innovative use of carbon nanotubes and bio-graphene [45] to enhance the performance and sensitivity of glucose oxidase. By studying the structural and functional interactions between these nanomaterials and glucose oxidase, the biosensor achieves improved electrochemical properties for the detection of glucose. Through this integration, the biosensor demonstrates enhanced efficacy in accurately detecting glucose levels, making it a promising tool for various applications, particularly in biomedical and clinical settings for DM management.

#### 2.1.2. Electronic Layout of the NIGM Component

Incorporating the aforementioned biosensor into the DEMIGOD system necessitated the inclusion of a potentiostat circuit. The potentiostat circuit plays a pivotal role in facilitating the electrochemical measurements required for sensor operation [46]. It enables the precise control and manipulation of the electrical potential between the working (WE) and reference electrodes (REs), allowing for accurate monitoring of the electrochemical reactions occurring at the sensor interface. By providing a stable and controlled electrical environment, the potentiostat circuit ensures reliable and reproducible sensor performance, essential for obtaining accurate and meaningful GM data. Thus, the integration of a potentiostat circuit is fundamental to harnessing the full potential of biosensors for glucose sensing applications [47,48,49].

In pursuit of developing a cost-effective closed-loop system for DM management, a meticulous selection process led to the adoption of a microcontroller-based potentiostat. This choice stemmed from the need for a versatile and compact solution capable of interfacing seamlessly with the biosensor while maintaining affordability and portability. Leveraging the programmability and computational power inherent in microcontrollers, the selected potentiostat was the Rodeostat FeatherWing +/−10 uA (source: https://iorodeo.com/products/rodeostat-feather-wing?variant=31618899607667 (accessed on 14 May 2024), Rodeostat FeatherWing +/−10 uA), tailored to meet the specific requirements of the biosensor, ensuring optimal electrochemical performance. The Rodeostat FeatherWing is an open-source potentiostat expressly devised to integrate seamlessly with Adafruit’s Feather/FeatherWing ecosystem. It is tailored to fit standard FeatherWing sockets and aligns with Feather Development boards adhering to Adafruit’s Feather specification, featuring analog output (DAC) on pin A0. Notably, the Rodeostat FeatherWing potentiostat offers an output range spanning +/−0.65 V, while the chosen variant for current measurement encompasses a range of +/−10 uA, meticulously selected to align with the requisites of the Alatzoglou C. et al [44] biosensor.

The selection of the Adafruit FeatherWing microcontroller was predicated upon the precise specifications of the potentiostat, particularly its voltage and current requirements. The Rodeostat FeatherWing potentiostat, boasting a nominal output range of +/− 1.65 V and a current measurement variant spanning +/− 10 uA, necessitated a microcontroller platform capable of accommodating these specifications while ensuring seamless integration and functionality. In this context, the Adafruit FeatherWing system emerged as a fitting choice due to its compatibility with the aforementioned potentiostat parameters.

The Adafruit Feather M0 Bluefruit LE microcontroller (source: https://learn.adafruit.com/adafruit-feather-m0-bluefruit-le/overview (accessed on 14 May 2024) Adafruit Feather M0 Bluefruit LE Overview) system aligns with the requirements of the Rodeostat FeatherWing potentiostat by offering a flexible platform with analog output (DAC) on pin A0, as specified. This compatibility enables the potentiostat to interface effectively with the microcontroller, facilitating the implementation of control algorithms for closed-loop GM. Additionally, the FeatherWing system’s modular design and adherence to open-source principles provide versatility and ease of integration, further underscoring its suitability for accommodating the precise specifications of the potentiostat. Thus, the selection of the Adafruit FeatherWing microcontroller system was driven by its alignment with the voltage and current requirements of the potentiostat, ensuring seamless integration and optimal performance within the closed-loop system for diabetes management. By integrating the potentiostat within a microcontroller platform, the closed-loop system gains the ability to execute real-time control algorithms for automated decision making, enhancing DEMIGOD’s responsiveness and adaptability to fluctuating glucose levels.

The interface between the Rodeostat FeatherWing potentiostat and the Adafruit Feather M0 Bluefruit LE development board is as follows:

- The voltage regulation between the working and reference electrodes is achieved through the utilization of analog output A0.
- The working electrode current (obtained from the transimpedance amplifier output) is acquired through the analog input A2.
- The voltage difference between the working electrode and the reference electrode is acquired at analog input A4.
- The counter electrode connect/disconnect switch is controlled using digital output D13. This can optionally be changed to D10, D11, or D12.

The Adafruit Feather M0 Bluefruit LE is a comprehensive development board, designed to integrate with the Arduino Integrated Development Environment (IDE) and leverage BLUETOOTH^®^ Low Energy technology. Equipped with a built-in USB port facilitating both data transfer and battery charging, this board features an ATSAMD21G18 ARM Cortex M0 processor operating at a clock speed of 48 MHz and operating at 3.3 V. The development board chip is endowed with 256 K of flash memory and 32 K of RAM, ensuring ample storage and computational capabilities for diverse project requirements. Moreover, the built-in USB interface facilitates USB-to-Serial programming, facilitating debugging processes.

In consideration of wearable applications, the Adafruit Feather M0 Bluefruit LE development board accommodates portable projects through the inclusion of a slot for Adafruit 3.7 V lithium polymer batteries, enabling untethered operation. Additionally, the board integrates the Bluefruit BTLE module alongside two status LEDs, facilitating seamless data transmission between the board and mobile devices via the requisite communication protocols utilizing UART services. This feature set renders the Adafruit Feather M0 Bluefruit LE particularly well- suited for mobile and web-based projects intended for deployment on smartphones or tablets. Furthermore, the board is compatible with various software environments, ensuring versatility across diverse computing platforms. In the developed prototype device presented in this manuscript, the connection between the Rodeostat FeatherWing potentiostat and the Adafruit Feather M0 Bluefruit LE microcontroller board was established using an Adafruit FeatherWing Doubler board (source: https://learn.adafruit.com/featherwing-proto-and-doubler/ (accessed on 22 October 2023), Adafruit FeatherWing Doubler board).

**Figure 2:**
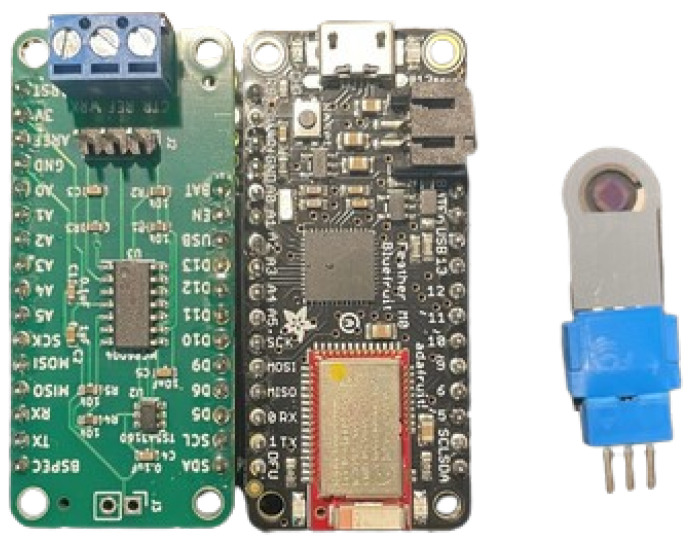
DEMIGOD NIGM system: Rodeostat FeatherWing +/− 10 uA potentiostat connected with the Adafruit Feather M0 Bluefruit LE microcontroller via an Adafruit FeatherWing Doubler board & SGM biosensor integrated with Amphenol FCI Clincher Connector.

The integration of Bluetooth connectivity enables the seamless transmission of data from the closed-loop system to a web-based application, thus facilitating remote monitoring and management of glucose levels. This wireless communication capability enhances the accessibility and convenience of the closed-loop system, allowing users to remotely monitor their glucose levels and treatment efficacy in real time [50]. Moreover, data transmitted via Bluetooth can be seamlessly integrated into the web-based software application, providing users with comprehensive insights into their glycemic trends and facilitating informed decision making regarding hyperglycemic treatment and lifestyle adjustments. To establish connectivity between the biosensor and the integrated system, an Amphenol FCI Clincher Connector (3 Position, Male) (source: https://www.hellasdigital.gr/electronics/connector/amphenol-fci-clincher-connector-3-position-male-com-14197/?sl=en (accessed on 22 October 2023), Amphenol FCI Clincher Connector) was employed as the connection tool. This connector interfaces with the potentiostat circuit board via cable jumpers. Overall, the incorporation of a microcontroller-based potentiostat system with Bluetooth connectivity represents a synergistic approach to the developed low-cost closed-loop system prioritizing both affordability and functionality in DM management (Figure 2).

#### 2.1.3. NIGM Component Software Development

The Rodeostat FeatherWing potentiostat, coupled with the Adafruit Feather M0 Bluefruit LE microcontroller, was programmed using Arduino IDE (version 1.8.19). Leveraging the functionalities offered by = Arduino IDE for software development, the glucose detection and data recording were implemented using C++ programming language. This integration seamlessly incorporates the potentiostat with the Adafruit Feather M0 Bluefruit LE, enabling robust communication and control protocols. Additionally, the programmed code facilitates chronoamperometric measurements, allowing the device to accurately assess electrochemical parameters. Furthermore, data acquisition and transmission to the web-based software application of the proposed system were facilitated through Bluetooth connectivity, ensuring real-time monitoring and analysis of experimental results.

### 2.2. Controlled Transdermal Nanoemulsion Release of Hypoglycemic Treatment

The objective of the HTRC component incorporated within the DEMIGOD system is to facilitate the automated, controlled administration of medication through noninvasive means, catering particularly to patients afflicted with DM, necessitating the precise and consistent regulation of glucose levels. Central to this component is a circuit utilizing thermal heating to activate gelatin pads.

Designed to attain a temperature of approximately 40 °C, the HTRC ensures a level of warmth deemed generally safe and nonhazardous for brief [51], indirect contact with human skin. Comprising a resistor ladder, the circuit establishes a predefined thin-layer grid, resulting in uniform heat distribution across the gelatin substrate, thus ensuring a consistent thermal response. The gelatin patch developed by M. Patila et al. [52], formulated as part of the HTRC, undergoes a phase transition upon exposure to the specified temperature range of approximately 37–40 °C, induced by the resistor array. This transition facilitates the controlled release of the encapsulated hypoglycemic substance.

With increasing heat, the viscosity of the gelatin diminishes, promoting a more fluidic environment. Consequently, the hypoglycemic treatment substance, uniformly dispersed within the gelatin matrix (5 patches—1 cm^2^ and 2.5% gelatin each patch), commences controlled diffusion. The mesh-like configuration of the resistance ladder ensures uniform release across the entire patch surface, mitigating localized concentration peaks and ensuring consistent delivery of the hypoglycemic agent.

#### 2.2.1. Electronic Layout of the HTRC Component

The HTRC encompasses the following elements:

- Arduino Nano: A compact, comprehensive, and breadboard-compatible board, the Arduino Nano is built upon the ATmega328 microcontroller architecture (Arduino Nano 3.x).
- HC-05 Bluetooth Wireless Serial Port Module: Facilitating wireless communication, the HC-05 Bluetooth module enables seamless serial data transmission between devices.
- LM35 Temperature Sensor: The LM35 sensor provides accurate and reliable temperature measurements, essential for monitoring thermal conditions within the system. In the context of the hypoglycemic release system circuit, the LM35 sensor was chosen for its suitability and efficacy.
- Resistor Ladder: The resistor ladder comprises a network of resistors designed to create a predefined thin-layer grid, ensuring uniform heat distribution across the gelatin substrate, thereby facilitating consistent thermal response.

The relationship between resistance and temperature is typically determined using the Steinhart–Hart equation:

$$\frac{1}{T}=A+B\ln\left(R\right)+C{\left(\ln\left(R\right)\right)}^{3}$$

In the envisaged configuration, the heating mechanism responsible for the dispensation of hypoglycemic substance is realized through the utilization of base resistors. These resistive components generate thermal energy as a consequential effect of the electrical current traversing through them, in accordance with Joule’s first law. This foundational law articulates that the power dissipation, denoted as *P*, within a resistor is directly proportional to the square of the current *I* flowing through it and the resistance *R* of the resistor, as expressed by the equation

$$P=I^{2}\times R$$

Considering the circuit’s prescribed threshold for maximum permissible heating, the dissipated power assumes a critical role in elevating the temperature of both the resistor itself and the contiguous environment, including any surface interfacing with the resistor. Through deliberate selection of resistors characterized by appropriate values and their arrangement either in series or in parallel configuration, the attainment of the desired power output, and hence the requisite thermal energy, can be effectively orchestrated.

A power supply, integrated with a regulating apparatus, exemplified by the TIP120 transistor under the control of an Arduino Nano in the current instantiation, exhibits the capacity to modulate the electric current traversing the resistors to sustain the designated temperature. This procedural approach is an efficacious and comparatively uncomplicated methodology for crafting a meticulously controlled heating interface. It is imperative to exercise diligence in the selection of resistors capable of withstanding the power dissipation they are subjected to, thereby averting potential damage or operational inefficiencies. In the scenario elucidated, addressing overheating concerns is tactically resolved by redistributing power dissipation across multiple resistive elements.

The resistors utilized exhibit a power rating of ½ Watt, and a configuration of 330 Ω × 9 is theoretically employed to mitigate cumulative errors over extended operational durations. The circuitry of the hypoglycemic therapy release system integrates an Single Pole, Double Throw (SPDT 1) miniature switch as the principal control interface. This component furnishes a manual means to engage or disengage the circuit, affording users an intuitive control over its functionality. Augmenting user interaction with immediate visual cues, the design incorporates LED indicators. A red LED illuminates upon circuit activation, signifying closure, whereas a distinct blue LED illuminates upon reception of a Bluetooth signal, facilitating the discernment of wireless command transmissions to the device.

**Figure 3:**
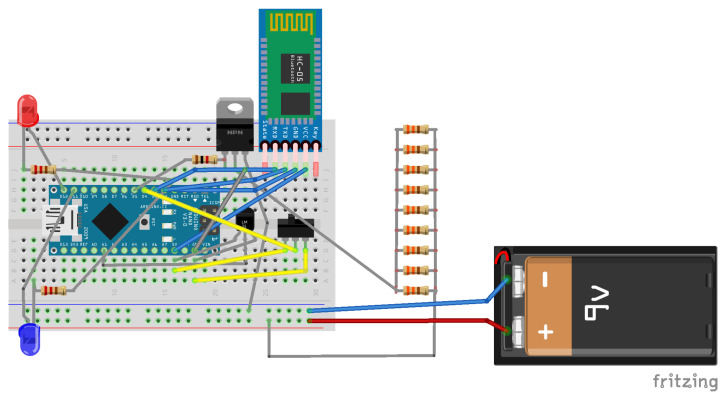
Circuit diagram of the controlled transdermal nanoemulsion release of hypoglycemic treatment system.

At the core of the circuit’s operational efficacy lies the LM35 temperature sensor, tasked with the continual surveillance of the heating surface’s temperature. This feedback loop serves as a pivotal mechanism, safeguarding against temperature fluctuations and preserving a consistent thermal environment. By virtue of this monitoring mechanism, the circuit maintains a steady temperature, thus averting the risk of overheating and sustaining the desired level of warmth across the heating surface. The schematic diagrams of the circuit (Figure 3) were created using Fritzing software (version 0.9.10) [53].

#### 2.2.2. HRTC Component Software Development

The programming code for the HRTC component was developed using the Arduino IDE (version 1.8.19), employing the C++ programming language. Leveraging the features provided by C++, the Arduino Nano microcontroller orchestrates precise control over the heating mechanism, thereby maintaining a stable temperature of approximately 40 °C for optimal hypoglycemic release from the gelatin pads. Moreover, integration with the HC-05 Bluetooth module enables seamless wireless communication between the system and its accompanying mobile application, facilitating real-time data transmission and remote monitoring and adjustment capabilities for users. In essence, by harnessing the capabilities of Arduino IDE and the versatility of C++, the heating circuit achieves a seamless integration of hardware control and wireless communication, ensuring the reliable and efficient operation of the release mechanism.

#### 2.2.3. Design and Fabrication of 3D-Printed Cases for DEMIGOD System Components

**Figure 4:**
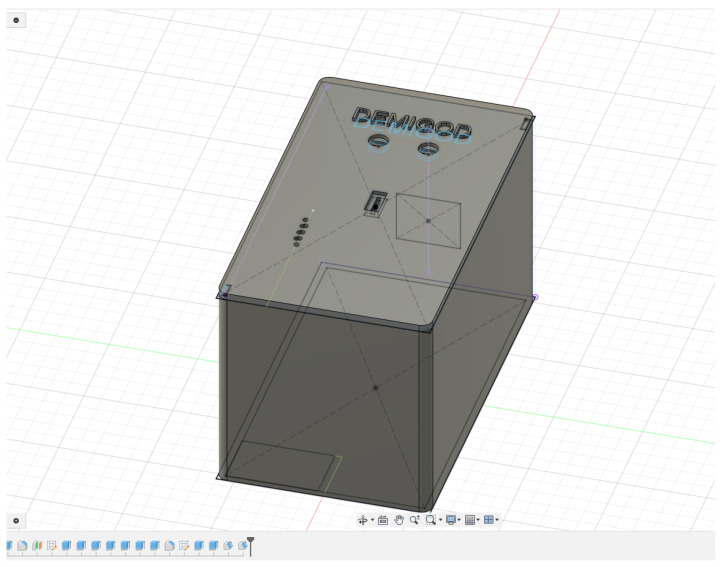
Three-dimensional design of the DEMIGOD case.

**Figure 5:**
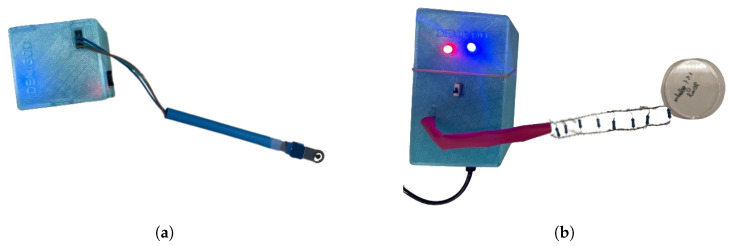
DEMIGOD device 3D-printed cases of components. (**a**) The 3D-printed case of the NIGM component. (**b**) The 3D-printed case of the HTRC component.

To ensure robust shielding and tailored accommodation for individual components, bespoke cases were devised and manufactured employing AutoCAD 2021 v24.0 for design purposes (Figure 4). Subsequently, Cura v5.4.0 slicer was utilized to generate G-code files compatible with the 3D printer, Creality Ender 3. These intricately crafted cases (Figure 5) serve to safeguard the circuits against environmental elements while offering a streamlined and space-efficient design, facilitating seamless integration and operational ease within their designated applications.

### 2.3. Web-Based Software Application for Personalized DM Care

**Figure 6:**
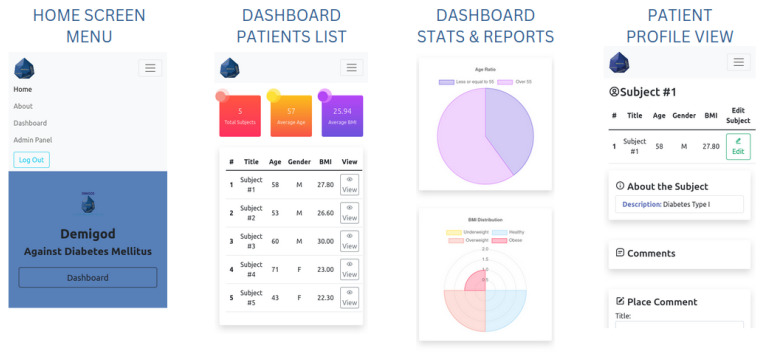
Web-based software application for personalized DM care views.

The devised web-based software application (Figure 6) facilitates real-time GM and remote activation of the HTRC via wireless interaction with the prototype device constructed. Central to the application’s architecture is Django technology, a robust Python 3 web framework renowned for its expeditious software development capabilities and streamlined design principles. Leveraging Django not only ensures the seamless operation of the application but also fortifies its security infrastructure, safeguarding sensitive health data from potential breaches.

To optimize user experience across a spectrum of devices, the application interfaces were crafted using Bootstrap technology. This adaptive framework tailors layout, graphics, and usability elements dynamically, ensuring a consistent and intuitive interface irrespective of the device’s form factor. The efficacy of the application lies in its ability to present data and outcomes in an interactive and comprehensible manner. To this end, Chart JS technology was employed for crafting visually appealing and flexible data visualizations. These graphical representations offer users immediate insights into their health metrics, empowering informed decision-making.

Ensuring the reliability and integrity of stored user health data and system metrics is paramount. Therefore, SQLite3, a lightweight yet potent database engine, was adopted for its swift data access, seamless updates, and robust security measures. A notable feature of the developed web-based software application is its real-time wireless communication capability with the proposed prototype device components. This functionality is achieved through integration with the pybluez package, a Python module facilitating Bluetooth communication. Consequently, users receive timely updates on their glucose levels directly within the application, facilitating prompt interventions as warranted.

### 2.4. Experiment Setup

**Figure 7:**
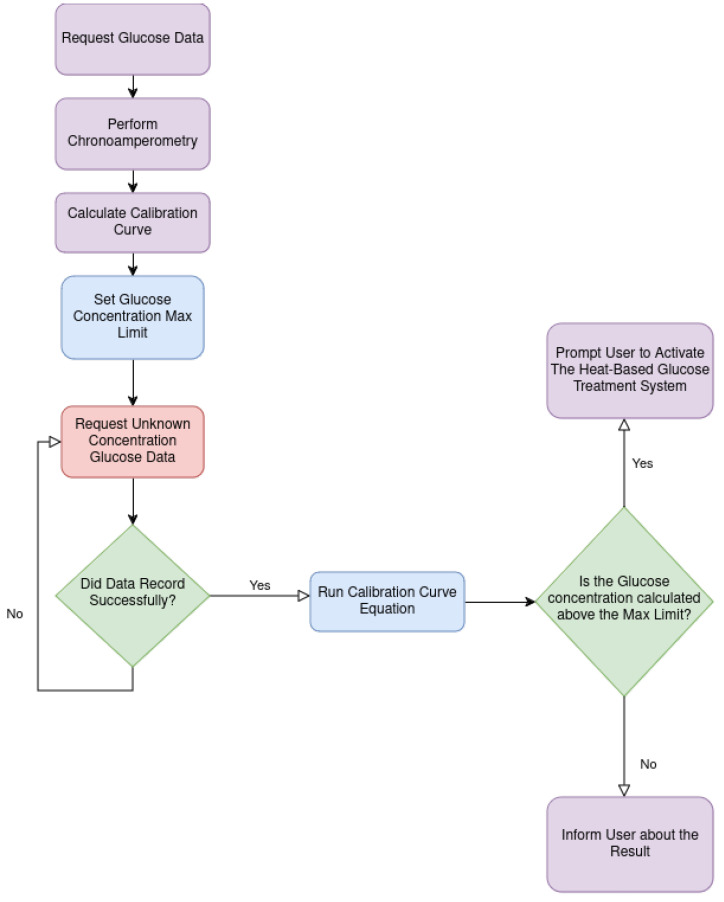
DEMIGOD system experimental flow chart.

**Figure 8:**
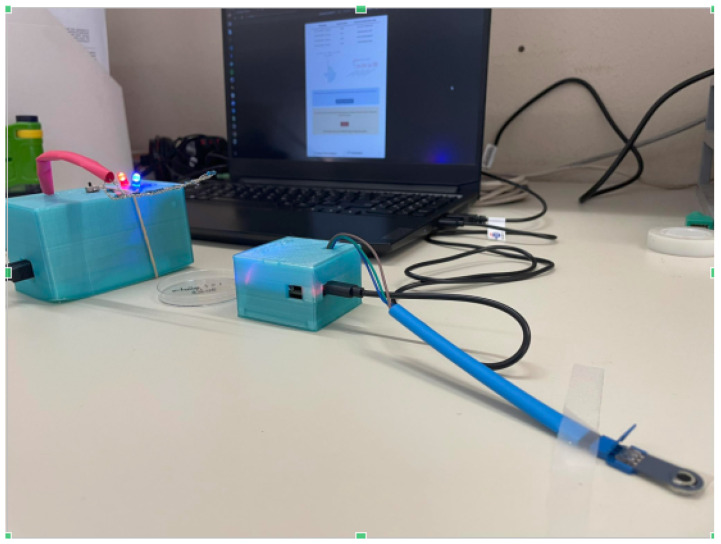
DEMIGOD system during the experimental procedure.

The experimental setup comprised two distinct segments, each meticulously conducted within a controlled laboratory environment. Initially, the focus was on the chronoamperometric test, which encompasses both the assessment of the DEMIGOD system’s decision-making mechanism based on the chronoamperometric analysis performed, as well as the system’s responsiveness to the web-based application interactions and the response of the HTRC component (Figure 7). We aimed to evaluate the proposed DEMIGOD system’s functionality and accuracy in detecting and responding to glucose fluctuations (Figure 8). Subsequently, the second category involved the in vivo hypoglycemic activity of nanoemulsion formulations. This procedure served as a means to evaluate the hypoglycemic impact of the nanoemulsions under investigation.

#### 2.4.1. Chronoamperometry Test and Response

The first segment of the experiments presented in this manuscript was conducted to investigate the response of the glucose detection system and decision making across a range of concentrations. The experiment entailed the recording of chronoamperometric signals, with recordings obtained at varying glucose concentrations. Chronoamperometry (CA) represents a time-dependent method involving the application of a square-wave potential to the working electrode. The resultant current, monitored over time, undergoes fluctuations, influenced by the diffusion of an analyte from the bulk solution toward the sensor surface. Consequently, CA serves as a valuable tool for assessing the current’s temporal variation, elucidating the diffusion-controlled processes transpiring at the electrode interface [54]. The concentrations tested included a blank control along with 10 μM, 50 μM, 100 μM, 200 μM, 400 μM, 600 μM, 800 μM, 1000 μM, 1200 μM, 1400 μM, and 1600 μM. The blank control solution serves as a reference or baseline solution devoid of the analyte under investigation. It typically consists of all the components of the experimental solution except for the analyte itself. The purpose of the blank control solution is to provide a comparison point for assessing the impact of the analyte on the system’s response. By measuring the baseline response in the absence of the analyte, researchers can distinguish between the signal attributed to the analyte and any background noise or interference present in the experimental setup.

The chronoamperometric measurements were performed over a duration of 30 s for each concentration level. During each recording session, the sensor was immersed in the appropriate glucose solution, and the chronoamperometric technique was employed to measure the current response generated by the sensor as a function of time. The experimental setup ensured precise control over the testing conditions, including temperature, humidity, and solution preparation, to maintain consistency and reliability throughout the experimentation process. The recorded chronoamperometric data provided insights into the sensor’s performance characteristics across a broad range of glucose concentrations, enabling a comprehensive evaluation of its detection capabilities. Wireless transmission of the detected signals to the software application via Bluetooth ensued, facilitating real-time data capture, processing, and analysis. Upon detection of sweat glucose concentrations surpassing 400 μM, the system initiated the hypoglycemic therapy release circuit at approximately 40 °C.

#### 2.4.2. In Vivo Hypoglycemic Activity of Nanoemulsion Formulations

##### Synthesis of Sitagliptin and Dapagliflozin Nanoemulsions

Sitagliptin is a DPP4 inhibitor, belonging to a class of oral hypoglycemics that block the enzyme dipeptidyl peptidase-4. It is used in the treatment of diabetes mellitus and acts by stimulating insulin release. On the other hand, dapagliflozin is an SGLT-2 inhibitor that works by increasing glucose excretion through the inhibition of sodium-glucose transport protein 2 [55]. For the preparation of sitagliptin and dapagliflozin nanoemulsions, an oil phase (caprylic acid), a surfactant (Tween-20), and a cosurfactant (propylene glycol) were selected. Double-distilled water was used as the aqueous phase. Smix (surfactant: cosurfactant) was initially prepared by mixing Tween-20 with propylene glycol at 1:2 (*v*/*v*). In the next step, 5.5 mL of the Smix was added to 1.5 mL of preheated caprylic acid at 60 °C under magnetic stirring to obtain a clear mixture, and then the hypoglycemic compounds (100 mg) were dissolved in this mixture. Under gentle stirring, water (3 mL) was added dropwise to the Smix/caprylic acid mixture and left to allow equilibration between each addition of water to form a clear homogeneous sample. The mixture was then added to a sonication bath for 10 min to create the final nanoemulsions.

##### Glucose Challenge in SV129 Mice

An oral glucose tolerance test (OGTT) was conducted to assess the hypoglycemic impact of the nanoemulsions. Thirty (30) male SV129 mice were randomly divided into three groups: the control group (*n* = 10), which received only the nanoemulsion; the sitagliptin group (*n* = 10), which was treated with sitagliptin at a dose of 10 mg/kg body weight; and the dapagliflozin group (*n* = 10), which was administered dapagliflozin at a dose of 10 mg/kg body weight. The animals were fasted overnight, and 30 μL of the nanoemulsion (equivalent to the stock solution) was applied to their shaved neck skin 30 min before the oral glucose injection (2 g/kg body weight). Glucose levels were monitored at baseline (0 h, before glucose administration) and at 1, 2, and 3 h after injection using a commercially available glucose strip meter. Blood samples were collected from the mice’s feet using a needle. The area under the curve (AUC) was then estimated to quantify the total increased blood glucose during an OGTT. Experiments were conducted with humane care, adhering to the European Union directive for the care and use of laboratory animals (EEC Directive 2010/63/EU; University of Ioannina, Approval no. 8099/6-6-2023).

##### Statistical Analysis

The data are presented as mean ± standard deviation (SD). Statistically significant variances among data means were analyzed using one-way analysis of variance, while differences between groups at various time intervals were evaluated using Student’s t-test (SPSS version 20.0, Statistical Package for the Social Sciences software, SPSS, Chicago, IL, USA). A significance level of *p* < 0.05 was applied. GraphPad Prism 8 software was utilized for generating the associated figures.

## 3. Results

**Figure 9:**
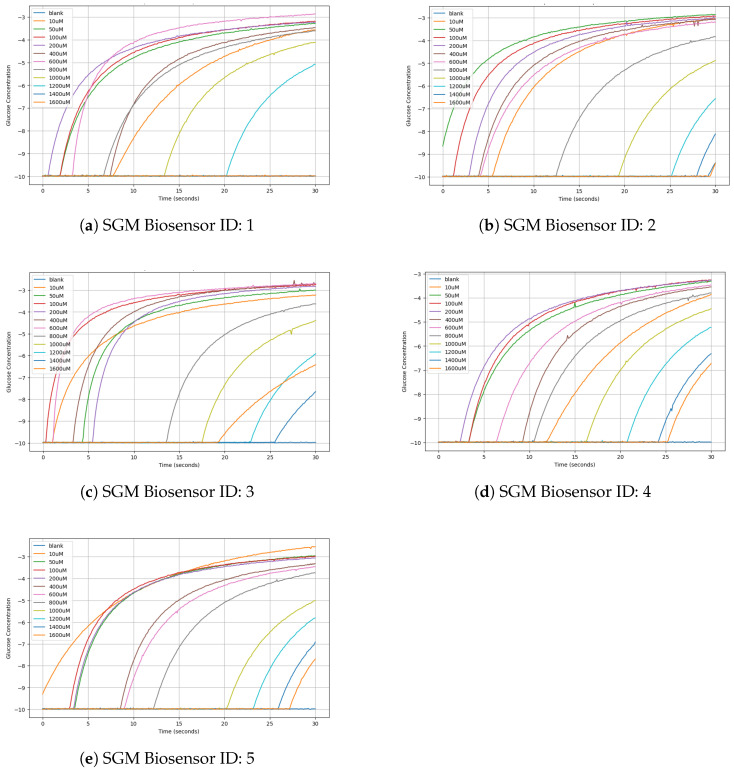
Chronoamperometry response over time for each SGM biosensor.

The results of the first experimental segment of the performed chronoamperometric measurements obtained using the NIGM component of the DEMIGOD prototype device are presented in Figure 9. Five distinct SGM Biosensors were tested (IDs 1–5), providing valuable insights into the performance and functionality of the NIGM component. These measurements present the NIGM’s dynamic range, sensitivity, and response time, elucidating its efficacy in detecting and quantifying different glucose concentrations. The chronoamperometric results (Table 1) provide a comprehensive understanding of the unique attributes and limitations of the NIGM component, providing valuable data for comparative analysis and optimization. Such detailed scrutiny not only facilitates the identification of optimal sensor configurations for noninvasive applications in GM but also informs future research endeavors aimed at enhancing the GM component’s performance and versatility.

**Figure 10:**
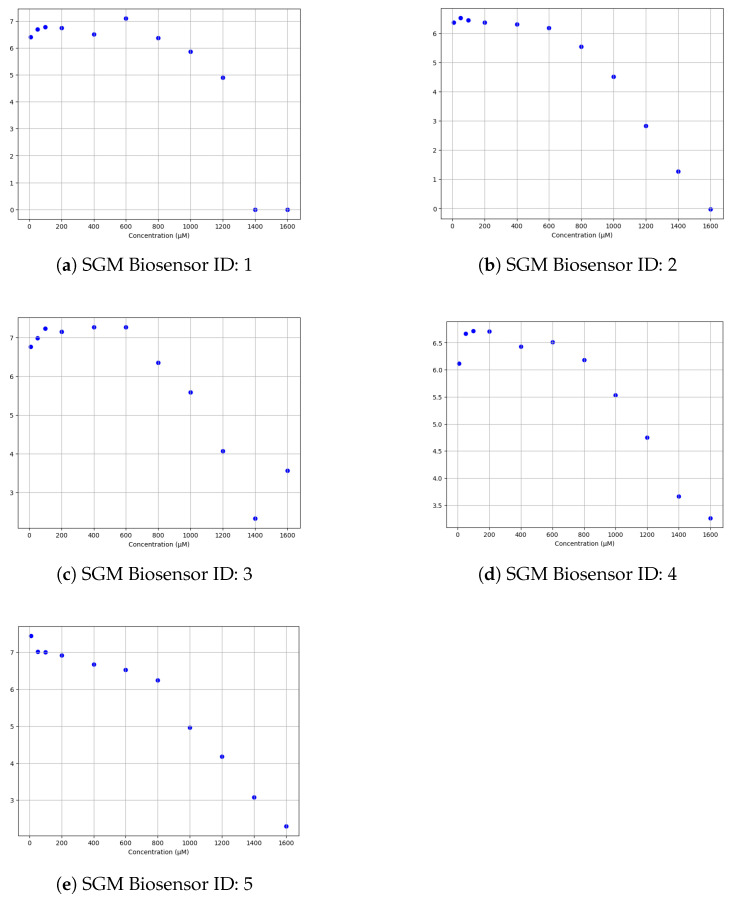
Calibration curves of each SGM Biosensor for each glucose concentration tested.

Utilizing the chronoamperometric results as a foundation, the calibration curve results (Table 2) were derived. To achieve this, the baseline current obtained from the blank solution devoid of the glucose solution was subtracted from the chronoamperometric results acquired at each known concentration level. This subtraction process effectively isolated the specific signal attributable to the analyte, enabling the construction of a calibration curve characterized by distinct concentration–response relationships, as presented in Figure 10 for each SGM Biosensor (IDs 1–5). With this calibrated framework in place, the DEMIGOD prototype system stands poised to accurately estimate the concentration of unknown glucose concentrations based on their corresponding chronoamperometric responses. By extrapolating from the established curve, the system holds promise for real-world applications, offering reliable glucose quantification capabilities for the noninvasive management of DM.

**Figure 11:**
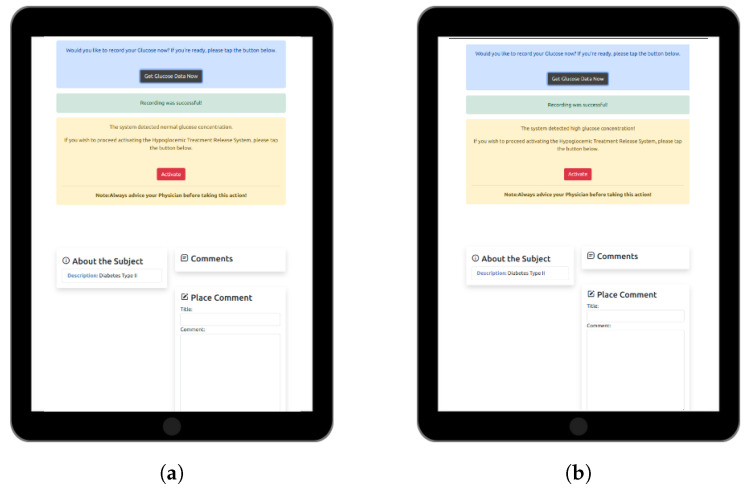
DEMIGOD system’s response to glucose concentrations as triggered by the web-based application. (**a**) Glucose concentration detected is below 400 μM. (**b**) Glucose concentration detected is above 400 μM.

**Figure 12:**
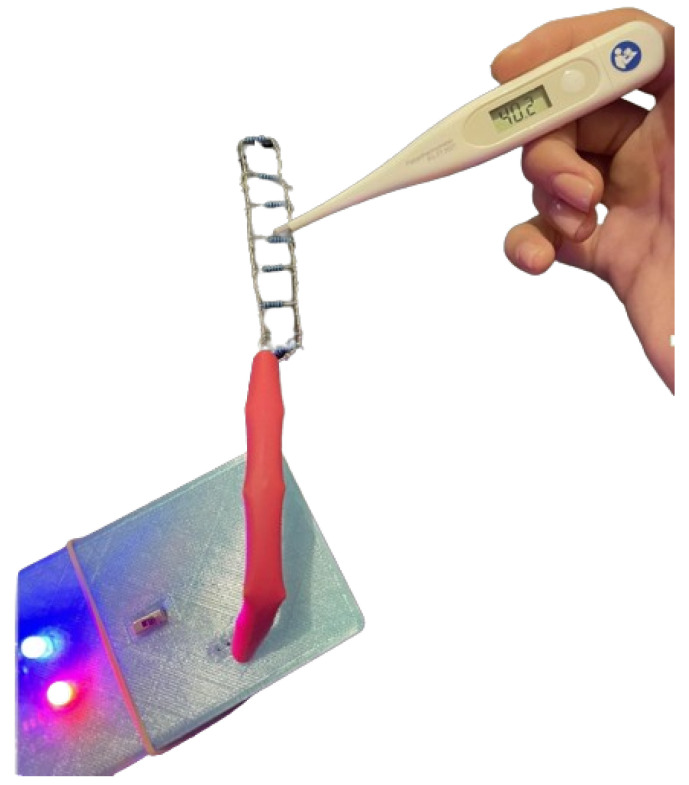
HRTC component triggered.

In response to varying glucose concentrations, the web-based application demonstrated appropriate reactions according to the following figures. When the glucose concentration was below 400 μM, the system exhibited specific responses, as depicted in Figure 11a, while when exceeding 400 μM, the system responded as shown in Figure 11b. These figures illustrate the system’s actions in maintaining glycemic control. The web-based interface effectively communicated with the system, triggering the HRTC component Figure 12. These results underscore the DEMIGOD system’s adaptability in responding to fluctuating glucose levels, ensuring optimal glycemic management across a range of conditions.

### Efficacy of Sitagliptin and Dapagliflozin Nanoemulsions

**Figure 13:**
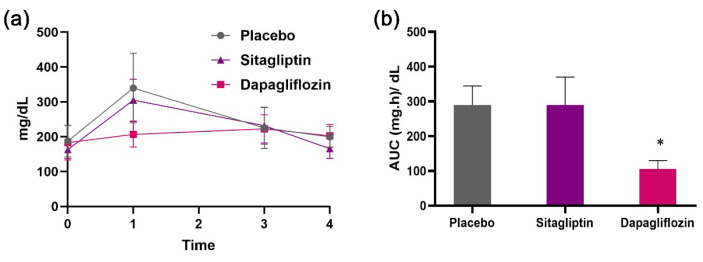
(**a**) Dapagliflozin nanoemulsion protected mice from a steep increase in blood glucose levels. (**b**) AUC was significantly lower in mice treated with nanodapagliflozin. Data are mean ± SEM; * *p* < 0.05 compared to vehicle (*n* = 10).

The intake of glucose significantly increased blood glucose concentrations in both the control and sitagliptin groups, rising from 188 ± 46 mg/dL to 340 ± 100 mg/dL and from 163 ± 25 mg/dL to 305 ± 60 mg/dL, respectively. However, in the dapagliflozin group, the increase in glucose was restricted, rising only from 183 ± 50 mg/dL to 207 ± 36 mg/dL. Glucose levels gradually returned to normal levels after 3 h (Figure 13a). The area under the curve (AUC) depicts the strong hypoglycemic effect of the dapagliflozin nanoemulsion (Figure 13b).

## 4. Related Work

During the last decade, great progress has been made not only from technological but also from regulatory points of view. Standard and approval agencies are taking NIGM devices seriously and are already setting guidelines for their approval and use in many countries [56]. For manufacturers and developers, this can seem cumbersome, as they have to adhere to stricter evaluation criteria. However, this also suggests that technology that seemed to belong only in the realm of science fiction is now possible, as evidenced by some devices currently on the market and others close to commercialization. All are based on different technologies that have evolved over the years.

Table 3 provides a comprehensive overview of various commercially available glucose testing instruments and artificial pancreas systems. It includes key information such as the brand, model, cost (in EUR), whether the device is invasive or noninvasive, and whether it provides insulin or other forms of therapy. The glucose testing instruments listed are commonly used by individuals with diabetes to monitor their blood glucose levels. In contrast, the artificial pancreas systems are advanced devices designed to automate blood glucose management by delivering insulin and, in some cases, other therapies. The costs listed are approximate and represent current prices; however, they may vary depending on the country, healthcare system, insurance coverage, and other factors. This comparison helps to highlight the range of options available to diabetes patients, from basic glucose monitors to sophisticated automated insulin delivery systems.

The DEMIGOD system presented in this manuscript addresses several gaps present in current commercial glucose testing instruments:

- Non-invasive monitoring: Unlike many commercial devices that require blood samples, our system uses a noninvasive sweat-based method, making it more user-friendly and suitable for continuous monitoring.
- Integrated decision making: The inclusion of a web-based application that interacts with the sensor system provides real-time monitoring and automated decision-making capabilities, which many commercial systems lack.
- Cost efficiency: As demonstrated in the cost analysis, our system offers a cost-effective alternative while maintaining high accuracy and reliability.
- Enhanced user experience: The web-based application offers a seamless user interface, remote monitoring capabilities, and integration with other health management systems, providing a comprehensive solution for glycemic control.

## 5. Discussion

Following the chronoamperometric measurements, calibration curves were constructed to elucidate the relationship between the glucose concentrations and the corresponding chronoamperometric responses. The calibration curve serves as a pivotal tool in the prediction process, empowering the web-based software application’s decision-making mechanism to discern the glucose concentration of unfamiliar samples. By correlating the chronoamperometric responses with known glucose concentrations, the calibration curve enables the system to extrapolate and interpolate values, providing reliable estimations even for untested concentrations. This calibration process underscores the importance of robust data analysis techniques in ensuring the precision and reliability of glucose concentration predictions within the system.

The chronoamperometric measurements revealed a discernible trend, showcasing a decrease in current intensity as the glucose concentration increased. Subsequently, the calibration curves were generated by subtracting the baseline current, obtained from the solution without glucose (blank) from each chronoamperometric result for every concentration. This adjustment ensured the isolation of the glucose-induced signal, enabling a precise evaluation of the sensor’s response to varying glucose levels. The resulting calibration curves exhibited a distinctive sigmoidal shape, reflecting the sensor’s sensitivity to changes in glucose concentration. Overall, the experimental findings underscore the efficacy of the system in accurately detecting and quantifying glucose levels, paving the way for its potential application in biomedical diagnostics and GM.

It is imperative to acknowledge several limitations inherent in the current study. Firstly, the sensitivity of the sensors utilized for glucose detection warrants further optimization to ensure accurate and reliable measurements, particularly within the dynamic range of physiological glucose concentrations. The regeneration capability of a three-electrode screen-printed SGM sensor can vary depending on several factors, including the materials used in the sensor construction, the sensing mechanism employed, and the operating conditions [72]. Moreover, variations in sweat quantity and composition among individuals pose challenges to consistent sensor performance, necessitating strategies for standardization and calibration. Additionally, while advancements have been made in enhancing the portability of the proposed noninvasive closed loop diagnostic and therapeutic system, there remains scope for refinement to enhance user convenience and usability in real-world settings.

## 6. Conclusions

The experimental endeavors presented in this manuscript underscore the promising potential of the proposed prototype device equipped with a nanoengineered noninvasive biosensor and its ability to provide the controlled transdermal nanoemulsion release of hypoglycemic treatment. Through the chronoamperometric measurements and calibration curve construction described in this manuscript, the device’s efficacy in accurately detecting and quantifying glucose across a wide range of concentrations was demonstrated. The evaluation of the NIGM component’s performance provided invaluable insights into its sensitivity, response dynamics, and versatility, paving the way for informed decisions regarding glucose detection and optimization. Furthermore, the high efficiency of the dapagliflozin nanoemulsion shows promise for alternative routes of diabetic drug administration. Moving forward, these findings lay a robust foundation for the continued refinement and deployment of sweat-based biosensor technology in biomedical diagnostics as well as for a novel therapeutic approach to managing DM.

In future work, efforts will be directed toward addressing the limitations identified in this study. Furthermore, the integration of data processing algorithms and decision-making mechanisms within the system requires rigorous validation to ensure their efficacy and safety. Tackling these limitations is crucial for advancing the translational potential of the proposed system and facilitating its successful implementation in clinical settings. By leveraging the capabilities of the DEMIGOD prototype device, we anticipate significant advancements in glucose detection and monitoring, ultimately contributing to enhanced public health efforts on a global scale.

## Figures and Tables

**Table 1:** Chronoamperometry results.

SGM Biosensor ID	Blank	10 μM	50 μM	100 μM	200 μM	400 μM	600 μM	800 μM	100 μM	1200 μM	1400 μM	1600 μM
1	−9.98	−3.57	−3.29	−3.2	−3.24	−3.47	−2.88	−3.61	−4.12	−5.09	−9.98	−9.98
2	−9.39	−3.02	−2.86	−2.94	−3.02	−3.08	−3.2	−3.84	−4.88	−6.56	−8.12	−9.41
3	−9.98	−3.22	3	−2.75	−2.83	−2.71	−2.71	−3.63	−4.39	−5.91	−7.65	−6.42
4	−9.98	−3.86	−3.31	−3.26	−3.27	−3.55	−3.47	−3.8	−4.45	−5.23	−6.32	−6.72
5	−9.98	−2.53	−2.96	−2.98	−3.06	−3.31	−3.45	−3.74	−5.01	−5.8	−6.91	−7.69

**Table 2:** Calibration curve results.

SGM Biosensor ID	10 μM	50 μM	100 μM	200 μM	400 μM	600 μM	800 μM	1000 μM	1200 μM	1400 μM	1600 μM
1	6.41	6.69	6.78	6.74	6.51	7.1	6.37	5.86	4.89	0.0	0.0
2	6.37	6.53	6.45	6.37	6.31	6.19	5.55	4.51	2.83	1.27	−0.02
3	6.76	6.98	7.23	7.15	7.27	7.27	6.35	5.59	4.07	2.33	3.56
4	6.12	6.67	6.72	6.71	6.43	6.51	6.18	5.53	4.75	3.66	3.26
5	7.45	7.02	7.0	6.92	6.67	6.53	6.24	4.97	4.18	3.07	2.29

**Table 3:** Cost analysis and comparison with commercial glucose testing instruments.

Brand	Model	Invasive	Provides Therapy	Cost (EUR)
Accu-Chek [57]	Guide	Yes	No	9.00
OneTouch [58]	Verio Reflect	Yes	No	25.89
Contour [59]	Next	Yes	No	13.79
FreeStyle [60]	Lite	Yes	No	23.91
iHealth [61]	Gluco+	Yes	No	36.79
Dario [62]	Blood Glucose Monitoring System	Yes	No	45.99
Keto-Mojo [63]	Blood Glucose & Ketone	Yes	No	56.85
ReliOn [64]	Platinum	Yes	No	18.48
CareSens [65]	N Premier	Yes	No	15.00
On Call [66]	Express II	Yes	No	18.39
Medtronic [67]	MiniMed 630G	Yes	Yes	7500
Medtronic [68]	MiniMed 770G	Yes	Yes	8000
Tandem [69]	t X2 with Control-IQ	Yes	Yes	7000
Insulet [70]	Omnipod 5	Yes	Yes	5000
Beta Bionics [71]	iLet Bionic Pancreas	Yes	Yes	9000
DEMIGOD (proposed system)	initial prototype	No	Yes	300

## Data Availability

The raw data supporting the conclusions of this article will be made available by the corresponding authors upon request.

## Abbreviations

The following abbreviations are used in this manuscript:

DM	Diabetes Mellitus
T1DM	Type 1 DM
T2DM	Type 2 DM
GM	Glucose Monitoring
SGM	Sweat-based Glucose Monitoring
CGM	Continuous Glucose Monitoring
SPE	Screen printed electrode
AID	Automated Insulin Delivery
NIGM	Non-Invasive Glucose Monitoring
HTRC	Hypoglycemic Treatment Release Circuit
WE	Working Electrode
RE	Reference Electrode
IDE	Integrated Development Environment
CA	Chronoamperometry
OGTT	Oral Glucose Tolerance Test
SD	Standard Deviation
